# Mediators of the effect of chiropractic care on 12- and 52-week outcomes for U.S. active-duty military personnel with low back pain: secondary analysis of a clinical trial

**DOI:** 10.1186/s12998-026-00628-0

**Published:** 2026-04-16

**Authors:** Zacariah K. Shannon, Cynthia R. Long, Robert D. Vining, Joan A. Walter, Ian D. Coulter, Christine M. Goertz

**Affiliations:** 1 Palmer College of Chiropractic, Davenport, IA USA; 2 Silver Spring, MD,, USA; 3 RAND Corporation, Santa Monica, CA USA; 4 Duke University School of Medicine, Durham, NC USA

**Keywords:** Pragmatic clinical trial, Chiropractic, Pain management, Back pain, Complementary therapies

## Abstract

**Background:**

The aim of this secondary analysis is to examine pain and non-pain factors as mediators of the effect of chiropractic care on 12- and 52-week outcomes from a clinical trial subsample of U.S. active-duty military personnel.

**Methods:**

Beginning in November 2014, the final 154 participants enrolled in a clinical trial, were asked to provide outcome data for 52 weeks. Data collection concluded in February 2016. We used natural effect models to evaluate 6-week change in the Roland-Morris Disability Questionnaire (RMDQ) and Numerical Pain Rating Scale (NPRS) as mediators of the effect of chiropractic care on 12- and 52-week outcomes of fatigue, sleep disturbance, and social role. We also evaluated non-pain factors as mediators of the effect of chiropractic care on pain outcomes. Models were adjusted by trial allocation minimization variables and by baseline values of the mediators and outcomes and results are reported as between-group differences on the outcome scales with 95% confidence intervals.

**Results:**

One hundred forty-four participants were included in the analysis. Participants had an approximate mean age of 33 years, were 78% male, and 63% white race. Except for NPRS, which demonstrated negligible difference at 52 weeks, total effects favored the group receiving chiropractic care. At 52-weeks the indirect effects through RMDQ were fatigue: 0.64 (95%CI −0.14 to 1.60), sleep: 0.82 (0.15 to 1.64), and social: 0.75 (0.12 to 1.65), mediating 44%, 17%, and 33% of the total effects. Indirect effects through non-pain factors on 52-week RMDQ were fatigue: 0.23 (−0.39 to 0.74), sleep: 0.20 (−0.32 to 0.80), and social: 0.38 (−0.06 to 0.94), mediating 12%, 10%, and 19% of the total effects.

**Conclusions:**

Improvement in pain intensity and pain-related disability during chiropractic care were drivers of 12- and 52-week sleep disturbance and social role outcomes for U.S. active-duty military. Mediation by fatigue, sleep disturbance, and social role of longer-term pain outcomes was small to negligible. A larger sample size and study of differing patient populations is needed to better understand how to tailor chiropractic care to target specific intermediates and achieve better patient outcomes.

**Trial registration:**

The trial was prospectively registered on clinicaltrials.gov (NCT01692275).

**Supplementary Information:**

The online version contains supplementary material available at 10.1186/s12998-026-00628-0.

## Background

Musculoskeletal low back pain (LBP) is well-recognized as a societal burden due to being highly prevalent [1], a leading cause of global disability [2, 3], and a top healthcare expenditure category in the U.S. [4]. Pain commonly impacts daily activities leading to a lowered ability to work [3] and lower overall life satisfaction [5, 6]. Though musculoskeletal pain affects daily life for people with varying occupations, high occupational physical demand is associated with higher odds of persistent pain [7]. 

One occupation with high physical demand and prevalence of persistent musculoskeletal pain is the military. Despite screening for spinal abnormalities upon entry into the military and programs aimed at maintaining physical fitness with training programs and fitness tests, musculoskeletal pain and injuries occur in half or more of active-duty U.S. military members [8]. Musculoskeletal pain and injuries are a leading reason for the inability to deploy to, and reason for evacuation from, combat theaters [8]. Early treatment with nonpharmacologic approaches is associated with a lower likelihood of military duty limitation, however [9]. 

Clinical practice guidelines in the U.S. recommend first-line treatment of LBP should be comprised of evidence-based nonpharmacologic approaches for acute and chronic low back pain [10]. Recommendations are strong for initial treatment with numerous physical, psychological, and combined physical-psychological interventions [11] due to greater anticipated treatment benefit than risk. However, the evidence supporting the efficacy of individual interventions is commonly categorized as low quality [10]. Some current research gaps for interventions recommended in guidelines include evidence of effectiveness when delivered in real-world clinical practice settings and limited duration of follow up.

Chiropractic care is a nonpharmacologic approach that is commonly sought for LBP treatment [12]. However, few trials have been conducted to examine chiropractic care for military members [13]. This work is a secondary analysis of a pragmatic clinical trial which previously reported greater improvement after 12 weeks for participants receiving chiropractic care plus usual medical care compared to receiving usual medical care alone for the primary outcomes of pain-related disability and intensity [14] and secondary physical, mental, and social health outcomes [15]. 

Secondary analyses of clinical trial data can provide important context to primary trial results. Mediation analysis is one approach to contextualize clinical trial results. Mediation analyses typically quantifies three effects: (1) the indirect effect defined as the effect of an exposure on an outcome that occurs through mediators of interest (2) the direct effect defined as the effect of an exposure on an outcome that does not occur through mediators of interest (3) the total effect defined as the total effect of an exposure on an outcome which is the sum of the indirect and direct effects [16]. Modeling these effects quantifies the contribution of intermediate factors to an overall effect. Mediation analysis can be useful for evaluating how targeting intermediate factors drives subsequent outcomes and, depending on the study design and research question, which intermediates may be targeted to improve outcomes or which intermediate factors targeting may be abandoned due to a lack of mediation effect [16]. A better understanding of intermediate factors and application through a targeted care approach has the potential to improve the effectiveness of care approaches.

Though mediation analysis can contribute to a better understanding of interventions, mediating factors of chiropractic care are not well known. A prior path analysis demonstrated that expectation of improvement may be an important component of improvement due to chiropractic care [17]. For other nonpharmacologic interventions, a broad range of factors have emerged as mediators. Pain intensity, physical activity, sleep, and self-efficacy have been shown to mediate stretching, yoga, physical therapy, and cognitive functional therapy [18–20]. Mediators of chiropractic care are likely similar to other nonpharmacologic interventions. Therefore, it appears important to explore mediating factors that span physical, mental, and social health domains.

The purpose of this work is to offer important context to the results of a pragmatic clinical trial studying chiropractic care for active-duty U.S. military members. We address limitations of prior work by exploring the role of intermediate, mediating factors on longer-term outcomes. The aim of this work is to estimate mediators of the effect of chiropractic care on 12- and 52-week pain, disability, fatigue, sleep disturbance, and social role outcomes for U.S. active-duty military personnel with low back pain in a pragmatic clinical trial.

## Methods

### Study registration and ethics approval

The clinical trial was approved by the RAND Corporation’s Human Subjects Protections Committee (# 2010 -782; last approval date: 10/10/2017) and the institutional review boards of Palmer College of Chiropractic (# 2010G137; last approval date: 11/22/2017), Walter Reed National Military Medical Center Bethesda (# 369462; last approval date: 11/16/2017), Naval Hospital Pensacola, Florida (# 384730; last approval date: 9/8/2016), and Naval Medical Center, San Diego (# 371029; last approval date: 10/14/2015). The trial was registered on clinical trials.gov (NCT01692275). Written, informed consent was obtained from each participant. An analysis plan for this secondary analysis was registered a priori (https://osf.io/w5yb8).

### Study design and source of data

This is a secondary analysis of data from a pragmatic clinical trial enrolling active-duty U.S. military personnel with LBP. This secondary analysis is exploratory and was not planned at the time of trial design. The trial protocol [21] and primary [14] and secondary outcomes [15] have been published. The clinical trial was conducted at three military treatment facilities (MTFs): Walter Reed National Military Medical Center in Bethesda, Maryland, Naval Medical Center San Diego in San Diego, California, and Naval Hospital Pensacola in Pensacola, Florida. Participants were allocated to usual medical care plus 6 weeks of chiropractic care or usual medical care alone. The initial trial protocol included follow-up for 12 weeks after enrollment, with a protocol amendment extending follow-up to 52 weeks after enrollment for the final 154 participants enrolled. Initial trial enrollment began in September 2012, enrollment of the final 154 agreeing to provide longer-term follow-up began in November 2014, and data collection concluded in February 2016.

For this analysis, we used an imputed dataset with complete outcome data for 144 participants who provided outcome data at 12 weeks or later. The imputed dataset was generated for another longitudinal analysis and accounted for substantial missing data that occurred at weeks 26 and 40 follow-ups. The assessment completion rates at the timepoints of interest for this study were 96% at week 6, 85% at week 12, and 81% at week 52. The complete outcome dataset was generated by replacing missing values with predicted values from linear mixed-effects regression models weighted by the inverse probability of missingness [22] using the variables of time, group, site, sex, age, worst pain intensity, and LBP duration, and a stepwise selection process for baseline covariates of race, ethnicity, Roland-Morris Disability Questionnaire (RMDQ) score, Numerical Pain Rating Scale (NPRS) score, and PROMIS-29 domain scores.

### Participants

Participants providing data for this analysis were enrolled at two MTFs: Walter Reed National Military Medical Center in Bethesda, Maryland, and Naval Hospital Pensacola in Pensacola, Florida. The primary method of recruitment was referral from MTF medical physicians. Inclusion criteria for trial participation included age between 18 and 50 years old and musculoskeletal LBP. Exclusion criteria included post-traumatic stress disorder diagnosis, recent spinal surgery, radiculopathy needing further evaluation, and conditions considered contraindications to spinal manipulation, including inflammatory arthropathy, instability, cauda equina syndrome, infection, fracture, or tumor.

### Sample size

This analysis was not planned at the time of trial design and is not powered. The original trial was powered by site, recruiting 250 participants per site (750 in total), to achieve the power to detect between-group differences for each site of 1.2 points in NPRS and 2.4 points in RMDQ [14].

### Effects of interest

The main effect of interest for this analysis is the indirect effect on the difference scale which is the estimate of the difference in effect between trial arms on the outcome that occurs through the mediator. The direct effect is the difference in effect between trial arms that does not occur through the mediator. The total effect is the difference between trial arms on the outcome and is equivalent to summing the indirect and direct effects. From these effect estimates, we also estimate the proportion mediated which is the proportion of the total effect estimated to occur through the mediator and calculated by dividing the indirect effect by the total effect.

### Measurement

#### Intervention

Participants in both trial arms received usual medical care and one trial arm was also allocated to receive up to 12 visits within 6 weeks with a chiropractic clinician at the MTF. Interventions for both usual medical care and chiropractic care were delivered as normal without alteration or direction from a trial protocol. A description of interventions delivered during the trial has been published [23]. Chiropractic care commonly included a combination of active and passive interventions with 77% of participants receiving an active intervention component and 94% receiving a passive intervention component [23]. The most frequent interventions delivered were spinal manipulation, exercise, and hot/cold packs [23] which are all recommended as first-line treatment for LBP in U.S. clinical practice guidelines [10]. 

We classify the intervention for this secondary analysis by the trial arm to which participants were allocated. Classifying intervention group by allocation allows us to model cause of the intervention in our model and addresses confounding of the exposure-mediator and exposure-outcome relationships.

#### Outcomes

The outcomes of interest for these mediation analyses include a combination of primary and secondary outcomes from the original trial to determine the effect on participant health domains that include pain and disability outcomes with other physical and psychosocial outcomes. Outcome domains were chosen in an attempt to report on health domains consistent with, although not all encompassing of, the biopsychosocial model of health as described by Engel [24]. Our chosen outcomes are disability, measured using the 0–24 point Roland-Morris Disability Questionnaire [25], pain intensity, measured using a 0–10 numerical pain rating scale (NPRS) [26], and fatigue, sleep disturbance, and satisfaction with participation in social role measured using PROMIS-29 v1.0 [27].

PROMIS domains are summarized as T-Scores which are normalized to a general population mean at a T-Score of 50 with a standard deviation equal to 10 [28]. PROMIS physical function and social role were transformed by taking 100-(T-Score) to make a higher score represent worse health across all outcomes. We transformed scores for these domains to make the reporting easier to understand with greater consistency between outcomes. The PROMIS domains of pain interference and physical function were not evaluated due to overlap with RMDQ, and anxiety and depression were not evaluated due to few mental health symptoms reported by this sample.

The chosen outcome timepoints of interest are 12 and 52 weeks. Twelve weeks demarcates the end of follow-up for the entire 750 participant sample and the endpoint of the primary results reporting, while 52 weeks is the last follow-up that was added for a subset of participants. These timepoints were chosen to quantify the mediation of intermediate and longer-term chiropractic care outcomes. Exploring mediation of 12-week outcomes also provides insight into consistency between the subsample and full trial sample, while exploration of mediation of 12 and 52-week outcomes may indicate if specific factors are more important for intermediate or longer-term outcomes.

#### Mediators

Mediators of interest for RMDQ and NPRS outcomes are PROMIS fatigue, sleep disturbance, and social role. Mediators of interest for PROMIS fatigue, sleep disturbance, and social role are RMDQ and NRS. The goal of exploring mediation in this way was to quantify the magnitude of effect on non-pain outcomes mediated by effects on pain and vice versa. We chose the mediators at 6 weeks, which was the end of the chiropractic treatment period in the trial. The evaluation of 6-week mediators of 12- and 52-week outcomes addresses how change during an initial course of chiropractic care mediates outcomes following the care period.

#### Confounding

As part of the trial design, the prognostic variables of sex, age, LBP duration, and LBP intensity were balanced between treatment arms at each site using adaptive allocation. These variables were included in our models to account for factors causing assignment to intervention. We accounted for mediator-outcome confounding by including baseline values of the mediators and outcomes in our models.

### Causal assumptions

The causal assumptions for these analyses are graphically displayed in directed acyclic graphs in supplemental figures S1 and S2. An assumption of the models used is no effect of intervention on mediator-outcome confounders.

### Statistical methods

We calculated and report baseline descriptive statistics by treatment group. We used the CMAverse package [29] in R statistical software (version 4.3.0) [30] to model natural effects [31]. Natural effect modeling was conducted for each individual mediator. Estimates from natural effect models include total effect, indirect effect, and direct effect and were calculated on the difference scale which is the difference between trial arms, and the proportion mediated. A positive effect estimate favors the group receiving chiropractic care. We used 1000 bootstrap samples to generate standard errors and 95% confidence intervals. Exposure-mediator interaction terms were evaluated for model inclusion by comparing model fit.

## Results

One hundred fifty-four participants were enrolled to provide outcome data for 52 weeks. Four participants withdrew, and 6 were lost to follow-up prior to 12 weeks. One hundred forty-four participants who provided data at 12 weeks or later were included in the analysis. Baseline characteristics of the 144 participants are shown in Table 1. The subsample included participants with a mean age of 33 years, 63% white race, and 78% male. Duration of pain was evenly split between chronic and acute pain. Compared to the general population average, the health domains showing the greatest deficit at baseline were pain-related disability and pain interference. Baseline mean pain intensity was 4.3/10.

**Table 1:** Baseline characteristics of participants receiving usual medical care plus chiropractic care vs. usual medical care alone (*n* = 144)

	UMC + CC (*n* = 72)	UMC (*n* = 72)
Age, years, mean (range)	32.7 (18–49)	32.7 (18–50)
Male, n (%)	57 (79)	56 (78)
Race, n (%)		
White	44 (61)	46 (64)
Black or African American	16 (22)	18 (25)
Other/Unspecified	12 (17)	8 (11)
Hispanic or Latino	7 (10)	13 (18)
LBP Duration, n (%)		
Acute (< 3 months)	38 (53)	35 (49)
Chronic (≥ 3 months)	34 (47)	37 (51)
RMDQ, 0–24, mean (SD)	9.4 (6.0)	10.4 (5.7)
Pain Intensity, 0–10, mean (SD)	4.2 (1.7)	4.3 (1.9)
PROMIS, T-Score, mean (SD)		
Pain Interference	60.0 (7.6)	59.7 (7.5)
Physical Function*	56.5 (7.4)	56.8 (6.4)
Sleep Disturbance	55.3 (6.9)	53.8 (8.4)
Fatigue	51.7 (10.2)	50.7 (10.7)
Anxiety	46.2 (7.0)	46.2 (8.6)
Depression	44.4 (5.5)	43.4 (6.2)
Social Role*	53.8 (8.3)	55.1 (9.3)

UMC+CC, Usual medical care plus chiropractic care; UMC, Usual medical care alone; *Variable is transformed 100-(T-Score) to make higher values indicate poorer health for all PROMIS domains

The results of natural effects models for the outcomes of RMDQ and NPRS are shown in Table 2. The total effect difference for RMDQ favored the chiropractic care group at 12 and 52 weeks. The total effect on NPRS favored the chiropractic care group at 12 weeks, but the difference was negligible at 52 weeks. Indirect effect estimates for the mediating role of fatigue and sleep disturbance for the outcomes of RMDQ and NPRS were consistently small for both outcomes and timepoints. The indirect effect for mediation of RMDQ by social role was 0.38 (95% CI −0.06 to 0.94) which was 19% of the total effect. The estimate for the proportion mediated by social role of the total effect on NPRS increased from 25 to 46% between 12 and 52 weeks, which was attributable to a small indirect effect estimate at each timepoint making up a larger proportion of a diminished total effect.

Table 3 displays the effect estimates from the models of mediation by 6-week RMDQ and NPRS of the 12 and 52-week outcomes of fatigue, sleep disturbance, and social role. The largest total effect difference at 52 weeks was for sleep disturbance (4.89). The total effect estimate for social role decreased from 3.49 at 12 weeks to 2.29 at 52 weeks. Indirect effect estimate sizes were generally consistent between mediators of RMDQ and NRS for each outcome and timepoint. Some of the indirect effect 95% confidence intervals did not cross zero, such as for mediation of 52-week sleep disturbance by RMDQ (total natural indirect effect = 0.82, 95% CI 0.15 to 1.64) and by NPRS (total natural indirect effect = 0.64, 95% CI 0.05 to 1.49). However, in these cases the remaining direct effects were large and the proportion mediated was small, indicating that much of the total effect was not mediated.

**Table 2:** Natural indirect, direct, and total effects and proportion mediated by fatigue, sleep disturbance, and social role of the effect difference between trial arms on 12- and 52-week outcomes of RMDQ and NPRS

Outcome	Mediator	Indirect effect (95% CI)	Direct effect (95% CI)	Total effect (95% CI)	Proportion
RMDQ 12 weeks	Fatigue	0.15 (−0.20 to 0.53)	1.81 (0.36 to 3.34)	1.96 (0.50 to 3.51)	0.08
	Sleep	0.13 (−0.22 to 0.54)	1.84 (0.42 to 3.38)	1.96 (0.53 to 3.55)	0.06
	Social	0.35 (−0.07 to 0.94)	1.61 (0.26 to 3.12)	1.96 (0.67 to 3.49)	0.18
RMDQ 52 weeks	Fatigue	0.23 (−0.39 to 0.74)	1.74 (0.11 to 3.47)	1.97 (0.19 to 3.71)	0.12
	Sleep	0.20 (−0.32 to 0.80)	1.77 (0.14 to 3.45)	1.97 (0.28 to 3.75)	0.10
	Social	0.38 (−0.06 to 0.94)	1.60 (−0.05 to 3.18)	1.97 (0.31 to 3.68)	0.19
NPRS 12 weeks	Fatigue	0.05 (−0.09 to 0.19)	0.56 (-0.01 to 1.10)	0.61 (0.04 to 1.17)	0.09
	Sleep	0.02 (−0.07 to 0.11)	0.59 (−0.01 to 1.18)	0.61 (0.002 to 1.19)	0.03
	Social	0.15 (−0.03 to 0.40)	0.46 (−0.04 to 0.98)	0.61 (0.06 to 1.21)	0.25
NPRS 52 weeks	Fatigue	0.06 (−0.09 to 0.22)	0.22 (−0.42 to 0.99)	0.28 (−0.40 to 1.03)	0.20
	Sleep	0.06 (−0.10 to 0.27)	0.22 (−0.45 to 0.95)	0.28 (−0.40 to 1.03)	0.22
	Social	0.13 (−0.03 to 0.34)	0.15 (−0.54 to 0.87)	0.28 (−0.42 to 1.00)	0.46

RMDQ, Roland-Morris disability questionnaire; NPRS, numerical pain rating scale; Sleep, sleep disturbance; Social, satisfaction with participation in social role

**Table 3:** Total natural indirect effect, total natural direct effect, total effect, and proportion mediated by RMDQ and NPRS of the effect difference between trial arms on 12- and 52-week outcomes of fatigue, sleep disturbance, and social role

Outcome	Mediator	Indirect effect (95% CI)	Direct effect (95% CI)	Total effect (95% CI)	Proportion
Fatigue 12 weeks	RMDQ	0.61 (−0.02 to 1.50)	0.71 (−1.62 to 3.24)	1.32 (−1.01 to 3.81)	0.46
	NPRS	0.50 (−0.05 to 1.40)	0.82 (−1.67 to 3.57)	1.32 (−1.15 to 3.98)	0.38
Fatigue 52 weeks	RMDQ	0.64 (−0.14 to 1.60)	0.81 (−2.20 to 4.10)	1.45 (−1.52 to 4.68)	0.44
	NPRS	0.65 (-0.02 to 1.79)	0.80 (−2.21 to 3.61)	1.45 (−1.50 to 4.35)	0.45
Sleep 12 weeks	RMDQ	0.58 (0.08 to 1.33)	2.36 (0.32 to 4.41)	2.94 (1.01 to 4.94)	0.20
	NPRS	0.48 (−0.005 to 1.17)	2.46 (0.58 to 4.41)	2.94 (1.02 to 4.95)	0.16
Sleep 52 weeks	RMDQ	0.82 (0.15 to 1.64)	4.07 (1.81 to 6.30)	4.89 (2.65 to 6.96)	0.17
	NPRS	0.64 (0.05 to 1.49)	4.25 (2.21 to 6.28)	4.89 (2.83 to 6.93)	0.13
Social 12 weeks	RMDQ	0.98 (0.21 to 2.00)	2.51 (0.09 to 5.03)	3.49 (1.21 to 5.92)	0.28
	NPRS	0.94 (0.13 to 1.84)	2.54 (0.05 to 4.86)	3.49 (1.00 to 5.99)	0.27
Social 52 weeks	RMDQ	0.75 (0.12 to 1.65)	1.54 (−0.77 to 3.89)	2.29 (0.16 to 4.45)	0.33
	NPRS	0.68 (−0.004 to 1.45)	1.61 (−0.45 to 4.00)	2.29 (0.31 to 4.49)	0.30

RMDQ, Roland-Morris disability questionnaire; NPRS, numerical pain rating scale; Sleep, sleep disturbance; Social, satisfaction with participation in social role

## Discussion

In this exploratory mediation analysis, effects of chiropractic care on 12- and 52-week sleep disturbance and social role outcomes were mediated by change in RMDQ and NPRS during the course of chiropractic care, though mediation was a small proportion of the total effect. Change in fatigue and sleep disturbance during care did not appear to drive longer-term pain intensity and disability outcomes. Effects of chiropractic care on pain intensity and disability attributable to change in social role were small.

Our analysis approach quantified the mediation of 12- and 52-week outcomes by the change in intermediate factors measured at the conclusion of 6 weeks of chiropractic care. This evaluation is pertinent to mediation of the effect of chiropractic care as it was delivered in the trial. The diagnoses provided and interventions delivered by chiropractors during the trial have been previously published [23] and are generally consistent with what has been reported in surveys of chiropractors in general practice in the US [32]. Our results add context to the primary trial results, and quantify the degree to which health change during a trial of chiropractic care drives longer-term outcomes.

The total effect of chiropractic care on pain intensity diminished between the 12-week and 52-week follow-up. As a result of the diminishing total effect on pain intensity, mediation of the effect of chiropractic care on pain intensity by social role made up 25% of the total 12-week effect and 46% of the total 52-week effect despite similar indirect effect estimate sizes. A larger improvement in social role during chiropractic care has the potential to lead to improved pain intensity outcomes in the longer-term. Prior work finding that social functioning mediates the effect of catastrophizing on pain intensity [33] suggests a plausible pathway by which these effects may be possible. However, our approach does not directly address the question of which intermediate factors could be targeted to produce better patient outcomes and further testing of the underlying assumptions of these relationships is needed.

Future work incorporating other factors not collected during this trial may provide a better understanding of the importance of tailoring clinical interventions to target specific mediators and achieve better patient outcomes. Patient beliefs such as self-efficacy, fear, and catastrophizing have emerged as important mediators of a number of treatment approaches for pain including mindfulness [34], cognitive functional therapy [20], and yoga [18]. Though there may be overlapping biological mechanisms engaged with a variety of nonpharmacologic interventions, chiropractic care has traditionally emphasized physical treatments such as spinal manipulation and exercise, in contrast with approaches such as mindfulness which also emphasize psychological components.

Alternative analysis approaches could be used to evaluate mediation in participant subgroups. Though evaluating mediators for subgroups has practical appeal, it would be at greater risk of bias than our analysis approach that used the trial assignment to intervention to address confounding. Subgrouping by participant characteristics would require adjustment for factors that confound the exposure-mediator and/or exposure-outcome relationships, factors that caused the participant subgrouping and which have a causal relationship with the mediator/outcome. Delineating effects with subgrouping would not be feasible due to sample size limitations imposed by this trial subsample.

### Limitations

This study has several limitations. We analyzed data from a clinical trial subsample and therefore our analyses were not powered to detect meaningful differences. The dataset we used in these analyses had complete outcome data and was generated in previous work by replacing missing outcome data with predicted values using linear mixed effects models weighted by the inverse probability of missingness under the assumption of missing at random.

Findings from this sample of active-duty military members may not be transportable to the general population. The sample was restricted to participants 18–50 years of age and were predominantly male, as expected for U.S. active-duty military members. Another key difference between the general population and U.S. active-duty military members is a well-documented tendency for military personnel to avoid reporting mental health symptoms [35, 36]. Similar to previous studies involving U.S. military personnel, participants enrolled in this clinical trial reported few mental health symptoms, likely due to a combination of being a younger and relatively healthy sample and the associated stigma. As a result, this prevented a meaningful evaluation of mental health measures as mediators or outcomes. Pain beliefs were also not measured during the trial which may be important for understanding changes in pain for military personnel [37]. 

Direct clinical application of the study findings may be limited due to the constraints of the sample size and population. However, the initial exploration in this work can be expanded in future research. Areas needing additional study include the mediating role of patient beliefs on chiropractic care outcomes, mediators in diverse patient populations, and how intervention components of chiropractic care affect outcomes.

## Conclusion

Improvement in pain intensity and pain-related disability during chiropractic care were drivers of 12- and 52-week sleep disturbance and social role outcomes for U.S. active-duty military. Effects on fatigue, sleep disturbance, and social role showed negligible or small mediation of effect on longer-term pain outcomes. A larger sample size and study of differing patient populations is needed to better understand how to tailor chiropractic care to target specific intermediates and achieve better patient outcomes.

## Supplementary Information

Below is the link to the electronic supplementary material.

Supplementary Material 1

## Data Availability

Data analyzed in the current study are available from Palmer College of Chiropractic, on reasonable request.

## References

[1] GBD 2017 Disease and Injury Incidence and Prevalence Collaborators. Global, regional, and National incidence, prevalence, and years lived with disability for 354 diseases and injuries for 195 countries and territories, 1990–2017: a systematic analysis for the global burden of disease study 2017. Lancet. 2018;392(10159):1789–858. 10.1016/S0140-6736(18)32279-7. 30496104 10.1016/S0140-6736(18)32279-7 PMC6227754

[2] Institute for Health Metrics and Evaluation (IHME). Global Burden of Disease 2021: Findings from the GBD 2021 Study. Seattle, WA IHME. 2024. https://www.healthdata.org/research-analysis/library/global-burden-disease-2021-findings-gbd-2021-study

[3] Ferreira ML, de Luca K, Haile LM, et al. Global, regional, and National burden of low back pain, 1990–2020, its attributable risk factors, and projections to 2050: a systematic analysis of the global burden of disease study 2021. Lancet Rheumatol. 2023;5(6):e316–29. 10.1016/S2665-9913(23)00098-X. 37273833 10.1016/S2665-9913(23)00098-X PMC10234592

[4] Dieleman JL, Cao J, Chapin A, et al. US health care spending by payer and health Condition, 1996–2016. JAMA. 2020;323(9):863–84. 10.1001/jama.2020.0734. 32125402 10.1001/jama.2020.0734 PMC7054840

[5] Stålnacke BM. Life satisfaction in patients with chronic pain – relation to pain intensity, disability, and psychological factors. Neuropsychiatr Dis Treat. 2011;7:683–9. 10.2147/NDT.S25321. 22128253 10.2147/NDT.S25321 PMC3225342

[6] McNamee P, Mendolia S. The effect of chronic pain on life satisfaction: evidence from Australian data. Soc Sci Med. 2014;121:65–73. 10.1016/j.socscimed.2014.09.019. 25306411 10.1016/j.socscimed.2014.09.019

[7] Johansson MS, Hartvigsen J, Korshøj M, Jensen MT, Holtermann A, Søgaard K. The leisure time and occupational physical activity paradox in persistent musculoskeletal pain. Sci Rep. 2025;15(1):21806. 10.1038/s41598-025-05815-2. 40594213 10.1038/s41598-025-05815-2 PMC12217324

[8] Molloy JM, Pendergrass TL, Lee IE, Chervak MC, Hauret KG, Rhon DI. Musculoskeletal injuries and united States army readiness part I: overview of injuries and their strategic impact. Mil Med. 2020;185(9–10):e1461–71. 10.1093/milmed/usaa027. 32175566 10.1093/milmed/usaa027

[9] Larson MJ, Adams RS, Ritter GA, et al. Associations of early treatments for Low-Back pain with military readiness outcomes. J Altern Complement Med. 2018;24(7):666–76. 10.1089/acm.2017.0290. 29589956 10.1089/acm.2017.0290 PMC6065526

[10] Qaseem A, Wilt TJ, McLean RM, Forciea MA, Clinical Guidelines Committee of the American College of Physicians. Noninvasive treatments for Acute, Subacute, and chronic low back pain: A clinical practice guideline from the American college of physicians. Ann Intern Med. 2017;166(7):514–30. 10.7326/M16-2367. 28192789 10.7326/M16-2367

[11] Mind and Body Practices, Accessed NCCIH. August 27, 2025. https://www.nccih.nih.gov/health/mind-and-body-practices

[12] Gallup I. Gallup-Palmer College of Chiropractic Annual Report | Managing Neck and Back Pain in America. Palmer College of Chiropractic. Accessed March 5, 2023. https://www.palmer.edu/wp-content/uploads/2021/12/palmer-gallup-annual-report-2018.pdf

[13] Mior S, Sutton D, Cancelliere C, et al. Chiropractic services in the active duty military setting: a scoping review. Chiropr Man Ther. 2019;27(1):45. 10.1186/s12998-019-0259-6. 10.1186/s12998-019-0259-6 PMC6628474 31338157

[14] Goertz CM, Long CR, Vining RD, Pohlman KA, Walter J, Coulter I. Effect of usual medical care plus chiropractic care vs usual medical care alone on pain and disability among US service members with low back pain: A comparative effectiveness clinical trial. JAMA Netw Open. 2018;1(1):e180105–180105. 10.1001/jamanetworkopen.2018.0105. 30646047 10.1001/jamanetworkopen.2018.0105 PMC6324527

[15] Hays RD, Shannon ZK, Long CR, et al. Health-Related quality of life among united States service members with low back pain receiving usual care plus chiropractic care vs usual care alone: secondary outcomes of a pragmatic clinical trial. Pain Med. 2022;23(9):1550–9. 10.1093/pm/pnac009. 35060609 10.1093/pm/pnac009 PMC9434322

[16] Vander Weele T. Explanation in causal inference: methods for mediation and interaction. Oxford: Oxford University Press; 2015. p. 8.

[17] Haas M, Vavrek D, Neradilek MB, Polissar N. A path analysis of the effects of the doctor-patient encounter and expectancy in an open-label randomized trial of spinal manipulation for the care of low back pain. BMC Complement Altern Med. 2014;14:16. 10.1186/1472-6882-14-16. 24410959 10.1186/1472-6882-14-16 PMC3897979

[18] Sherman KJ, Wellman RD, Cook AJ, Cherkin DC, Ceballos RM. Mediators of yoga and stretching for chronic low back pain. Evid Based Complement Alternat Med. 2013;2013:130818. 10.1155/2013/130818. 23690832 10.1155/2013/130818 PMC3652191

[19] Joyce CT, Chernofsky A, Lodi S, Sherman KJ, Saper RB, Roseen EJ. Do physical therapy and yoga improve pain and disability through psychological mechanisms? A causal mediation analysis of adults with chronic low back pain. J Orthop Sports Phys Ther. 2022;52(7):470–83. 10.2519/jospt.2022.10813. 35584010 10.2519/jospt.2022.10813 PMC9510936

[20] Schütze R, Liew B, Caneiro JP, et al. Mechanisms of change in cognitive functional therapy: a longitudinal mediation analysis of the RESTORE clinical trial for disabling chronic low back pain. Behav Res Ther. 2025;193:104853. 10.1016/j.brat.2025.104853. 40925074 10.1016/j.brat.2025.104853

[21] Goertz CM, Long CR, Vining RD, et al. Assessment of chiropractic treatment for active duty, U.S. Military personnel with low back pain: study protocol for a randomized controlled trial. Trials. 2016;17(1):70. 10.1186/s13063-016-1193-8. 26857706 10.1186/s13063-016-1193-8 PMC4746780

[22] Schulz KF, Grimes DA. Sample size slippages in randomised trials: exclusions and the lost and wayward. Lancet. 2002;359(9308):781–5. 10.1016/S0140-6736(02)07882-0. 11888606 10.1016/S0140-6736(02)07882-0

[23] Ziegler AML, Shannon Z, Long CR, et al. Chiropractic services and diagnoses for low back pain in 3 U.S. Department of defense military treatment facilities: a secondary analysis of a pragmatic clinical trial. J Manipulative Physiol Ther. 2021;44(9):690–8. 10.1016/j.jmpt.2022.03.009. 35752500 10.1016/j.jmpt.2022.03.009

[24] Engel GL. The need for a new medical model: a challenge for biomedicine. Science. 1977;196(4286):129–36. 847460 10.1126/science.847460

[25] Roland M, Morris R. A study of the natural history of back pain. Part I: development of a reliable and sensitive measure of disability in low-back pain. Spine (Phila Pa 1976). 1983;8(2):141–4. 10.1097/00007632-198303000-00004. 6222486 10.1097/00007632-198303000-00004

[26] Childs JD, Piva SR, Fritz JM. Responsiveness of the numeric pain rating scale in patients with low back pain. Spine (Phila Pa 1976). 2005;30(11):1331–4. 10.1097/01.brs.0000164099.92112.29. 15928561 10.1097/01.brs.0000164099.92112.29

[27] PROMIS Health Organization and PROMIS Cooperative Group, March PROMIS. 27, 2023. Accessed December 18, 2023. https://www.healthmeasures.net/explore-measurement-systems/promis

[28] PROMIS Health Organization and PROMIS Cooperative Group. Intro to PROMIS. April 12. 2023. Accessed December 18, 2023. https://www.healthmeasures.net/explore-measurement-systems/promis/intro-to-promis

[29] Shi B, Choirat C, Coull BA, VanderWeele TJ, Valeri L. CMAverse: A suite of functions for reproducible causal mediation analyses. Epidemiology. 2021;32(5):e20–2. 10.1097/EDE.0000000000001378. 34028370 10.1097/EDE.0000000000001378

[30] R Core Team. R: A Language and Environment for Statistical Computing. Published online 2023. https://www.R-project.org

[31] Vansteelandt S, Bekaert M, Lange T. Imputation strategies for the Estimation of natural direct and indirect effects. Epidemiol Methods. 2012;1(1):131–58. 10.1515/2161-962X.1014.

[32] National Board of Chiropractic Examiners. Practice Analysis of Chiropractic. 2020. Accessed December 18, 2023. https://www.nbce.org/news/practice-analysis-of-chiropractic/

[33] Papianou LN, Wilson JM, Edwards RR, Sieberg CB, Meints SM. The mediating effect of social functioning on the relationship between catastrophizing and pain among patients with chronic low back pain. Pain Med. 2023;24(11):1244–50. 10.1093/pm/pnad093. 37399110 10.1093/pm/pnad093 PMC13032065

[34] Adamowicz JL, Grant A, Calvert C, et al. Differences among veterans with chronic overlapping pain conditions and other chronic pain: baseline results from the LAMP pain management trial. Eur J Pain. 2025;29(9):e70125. 10.1002/ejp.70125. 40948198 10.1002/ejp.70125

[35] Greene-Shortridge TM, Britt TW, Castro CA. The stigma of mental health problems in the military. Mil Med. 2007;172(2):157–61. 10.7205/milmed.172.2.157. 17357770 10.7205/milmed.172.2.157

[36] Schreiber M, McEnany GP, Stigma. American military personnel and mental health care: challenges from Iraq and Afghanistan. J Ment Health. 2015;24(1):54–9. 10.3109/09638237.2014.971147. 25587819 10.3109/09638237.2014.971147

[37] Karasel S, Cebeci D, Sonmez I. Chronic pain and pain belief in active military personnel: a Cross-sectional study. Med Arch. 2020;74(6):455–62. 10.5455/medarh.2020.74.455-462. 33603271 10.5455/medarh.2020.74.455-462 PMC7879343

