# Supplementary material for: Mediators of the effect of chiropractic care on 12- and 52-week outcomes for U.S. active-duty military personnel with low back pain: secondary analysis of a clinical trial
Source: Chiropr Man Therap. 2026 Apr 16;34:12. doi: 10.1186/s12998-026-00628-0 (PMC13088719; doi:10.1186/s12998-026-00628-0)
Supplement: Supplementary file 1 — Supplementary Material 1 [file 12998_2026_628_MOESM1_ESM.docx]

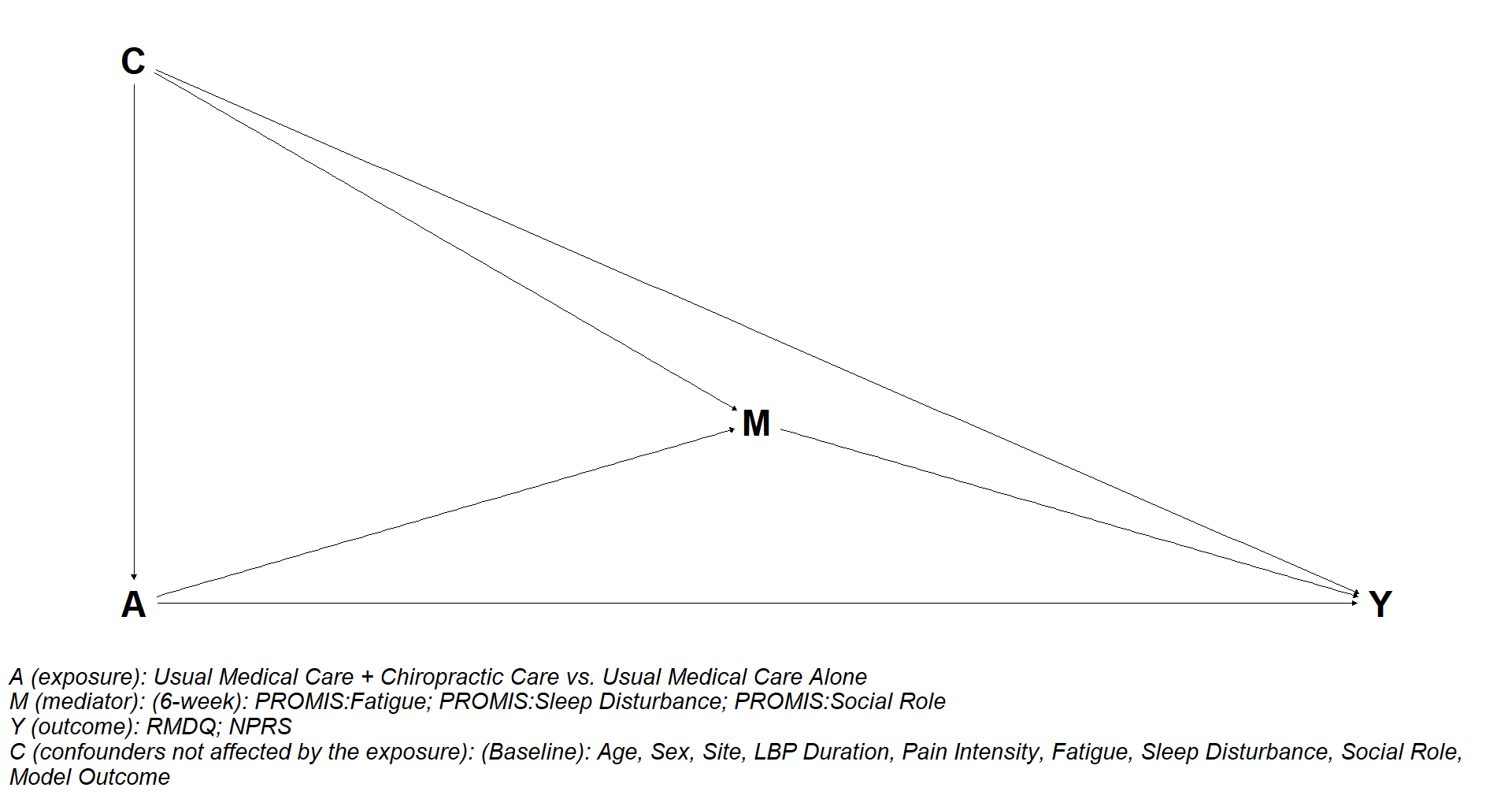


Figure S1. Directed acyclic graph for mediation of the effect of chiropractic care on 12- and 52-week RMDQ and NPRS.


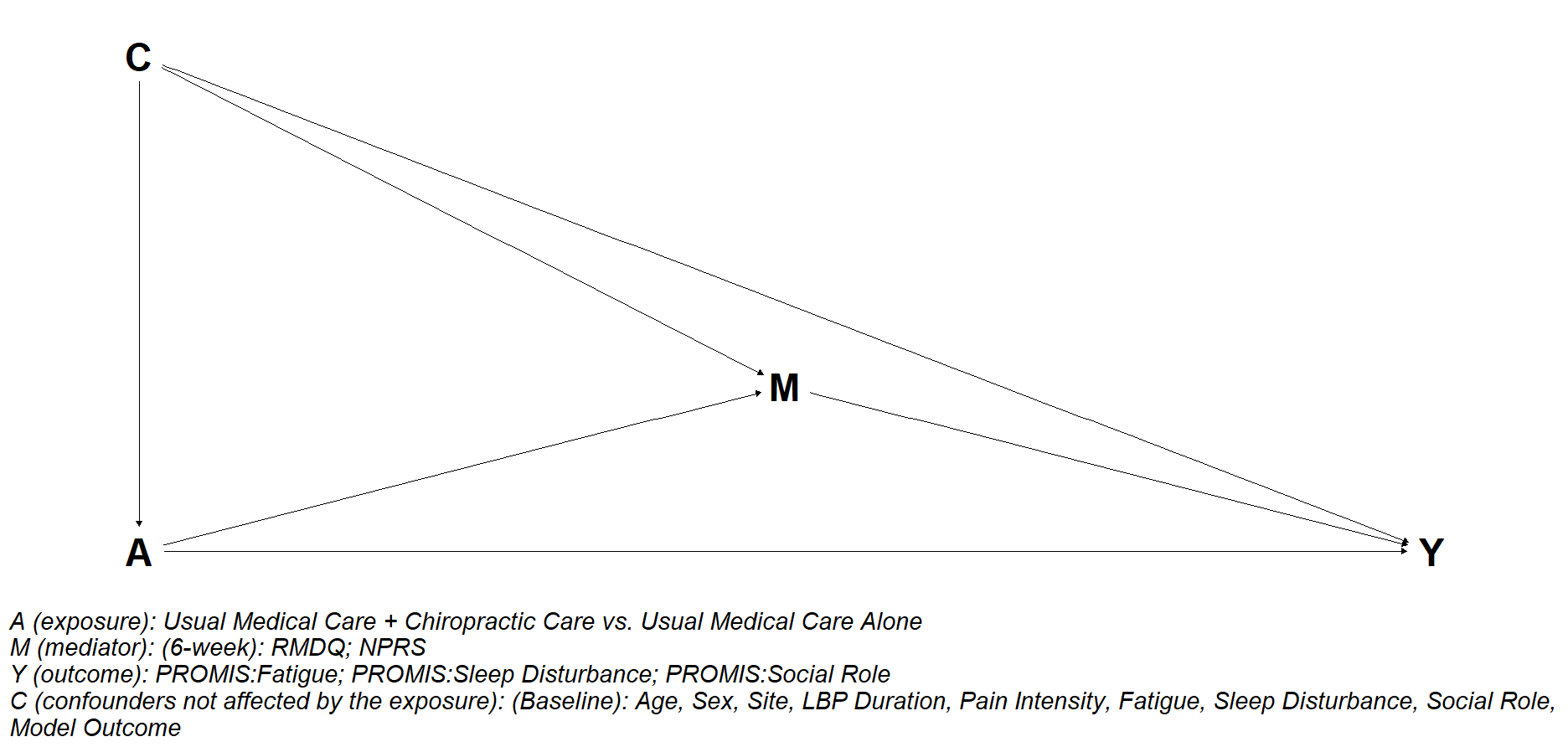


Figure S2. Directed acyclic graph for mediation of the effect of chiropractic care on 12- and 52-week PROMIS: fatigue, sleep disturbance, social role
